# ERK1/2 Signaling in Intrahepatic Cholangiocarcinoma: From Preclinical Advances to Therapeutic Strategies

**DOI:** 10.3390/biology14070776

**Published:** 2025-06-27

**Authors:** Veronica Porreca, Luca Sallustio, Ludovica Giancola, Pietro Angelone, Giuseppina Mignogna, Bruno Maras, Carmine Mancone

**Affiliations:** 1 Department of Molecular Medicine, Sapienza University of Rome, 00161 Rome, Italy; sallustio.1909220@studenti.uniroma1.it (L.S.); giancola.1853273@studenti.uniroma1.it (L.G.); angelone.1881277@studenti.uniroma1.it (P.A.); carmine.mancone@uniroma1.it (C.M.); 2 Department of Biochemical Science, Sapienza University of Rome, Piazzale Aldo Moro 5, 00185 Rome, Italy; pina.mignogna@uniroma1.it (G.M.); bruno.maras@uniroma1.it (B.M.)

**Keywords:** ERK1/2, intrahepatic cholangiocarcinoma, target therapy, personalized medicine, tumor microenvironment

## Abstract

Extracellular signal-regulated kinase 1/2 (ERK1/2) is involved in the regulation of the key cellular processes that are essential for the proper functioning of the cell under physiological conditions. Notably, the hyperactivation of ERK1/2 is implicated in oncogenesis and metastatic dissemination across various tumor types, making it an attractive candidate for targeted therapy (TT) through functional inhibition. In intrahepatic cholangiocarcinoma (iCCA), sustained ERK1/2 activation represents one of the major events within the complex signaling network that drives tumor development and progression. In this review, we dissect the biological role of ERK1/2 signaling in iCCA and highlight recent preclinical advances involving selective small-molecule ERK1/2 inhibitors. In vitro and in vivo studies have demonstrated how these inhibitors present effective anti-tumorigenic properties. In particular, PD901 and U0126 effectively reduce iCCA cell proliferation and invasion. Furthermore, Ulixertinib has shown a favorable therapeutic index and encouraging activity in clinical trials involving advanced solid tumors, including iCCA, paving the way for a new therapeutic approach targeting ERK1/2. Nevertheless, the heterogeneous and dynamic molecular landscape of iCCA, often accompanied by drug resistance, presents significant therapeutic challenges. We underscore how targeting the ERK1/2 pathway could represent a cornerstone within a multifaceted therapeutic strategy, fostering the development of personalized treatment approaches and improving clinical outcomes in iCCA patients.

## Simple Summary

Intrahepatic cholangiocarcinoma (iCCA) is a highly aggressive liver cancer with poor prognosis and limited treatment options. Its silent progression is a major hallmark, which delays diagnosis and limits therapeutic success. Extracellular signal-regulated kinase 1/2 (ERK1/2) signaling, which regulates critical cellular functions, is frequently hyperactivated in several types of tumors, including iCCA. Due to its central role in cancer progression, the ERK1/2 cascade has emerged as a promising candidate for target therapy (TT). This review discusses the role of ERK1/2 signaling and highlights recent pre-clinical advances that explore how small molecules can inhibit ERK1/2 pathway hyperactivation. In light of the growing interest in TT and personalized medicine (PM), ERK1/2 signaling emerges as a promising therapeutic target for counteracting iCCA aggressiveness.

## 1. Introduction

Intrahepatic cholangiocarcinoma (iCCA) is the second most prevalent primary liver cancer, following hepatocellular carcinoma (HCC) [1,2,3]. This heterogeneous and lethal hepatobiliary tumor originates proximally to the second-order bile duct and carries a high burden of morbidity and mortality. To date, iCCA shows a mortality rate exceeding 2 cases per 100,000 inhabitants per year in males [4]. Its aggressiveness is marked by the high proliferative and invasive capacity of malignant cholangiocytes, which rapidly disseminate to regional lymph nodes, largely driven by a abundant lymphatic vessel sprouting within the tumor microenvironment (TME) [5]. Despite the presence of multiple risk factors contributing to the development of iCCA, including primary sclerosing cholangitis (PSC), liver cirrhosis, and chronic hepatitis (B and C), early diagnosis remains a major challenge [6,7,8].

The asymptomatic nature of the disease in its first stages, its complex anatomical location, and its dense desmoplasia lead to late-stage detection and poor prognosis [9]. Surgical resection and liver transplantation represent the most effective curative option, although they are typically reserved for cases diagnosed at an early stage [8,10]. However, even after surgery, the five-year survival of patients undergoing liver resection varies only between 30% and 35%, with a median overall survival (OS) of approximately 28 months [8,11].

In modern oncology, target therapy (TT) and personalized medicine (PM) have emerged to address the limitations of traditional oncology treatments, such as chemotherapy, which often lack sufficient efficacy and may result in unwanted side effects. Both approaches aim to develop more effective and less toxic therapies, representing promising strategies in cancer treatment and prevention [12]. In this new therapeutic scenario, the advent of the “omics era” has equipped TT and PM with valuable tools to pinpoint specific therapeutic targets and develop personalized treatment for each patient [13,14]. This synergy marks a fundamental step towards precision medicine [15]. Specifically, while PM adopts a broader approach by integrating individual molecular, genetic, environmental, and lifestyle data to tailor therapies, TT focuses on selectively inhibiting specific proteins or signaling pathways that are involved in tumor growth and progression. As a cornerstone of PM, TT enhances efficacy and reduces toxicity by specifically targeting cancer cell abnormalities [16,17]. Depending on the molecular target, TT can act on surface antigens, growth factors, receptors, or intracellular signaling pathways that regulate cell proliferation, survival, metastasis, and angiogenesis [12].

Given the molecular complexity and aggressiveness of iCCA, omics and multi-omics approaches have been proven valuable in investigating the high intra- and inter-tumoral heterogeneity, leading to the identification of novel potential biomarkers for early diagnosis and potential therapeutic targets. These advancements are particularly relevant to overcome the challenges related to late-stage diagnosis and limited surgical options, particularly in the context of TT and PM [18]. This new scenario highlights the importance of identifying the key molecular targets involved in iCCA tumorigenesis and progression. Among these, preclinical studies have identified the extracellular signal-regulated kinase 1/2 (ERK1/2) signaling cascade as one of the key regulatory axes implicated in iCCA aggressiveness, chemoresistance, and immune modulation [14,19,20,21].

While ERK1/2 overactivation has been strongly associated with poor prognosis in several solid tumors [22,23,24], its clinical relevance in iCCA is currently under investigation [25,26,27]. Nevertheless, based on compelling preclinical evidence, the ERK1/2 pathway is being explored both for its prognostic value [28] and as a new potential therapeutic approach in iCCA management [27]. Upon activation by various extracellular microenvironment stimuli, including growth factors, hormones, cytokines, or stress signals, ERK1/2 acts as a critical regulatory hub, maintaining cellular homeostasis and facilitating the rapid and dynamic adaptation of the cell to extracellular cues [29,30,31]. ERK1/2 supports tumor cell proliferation and survival, while actively reshaping the TME. Persistent activation of ERK1/2 upregulates the expression of metalloproteases (MMPs), which facilitate tumor invasion and metastasis by degrading the extracellular matrix (ECM) [32]. Moreover, ERK1/2-driven MMPs expression recruits immune cells and promotes stromal remodeling, further enhancing the invasiveness of iCCA cells [28]. In light of the aforementioned background, this review investigates the role of the ERK1/2 signaling pathway in iCCA progression, emphasizes its molecular implications and therapeutic potential, and discusses the latest advancements in this research field.

## 2. Fundamental Insights into ERK1/2 Signaling Pathways

**Figure 1:**
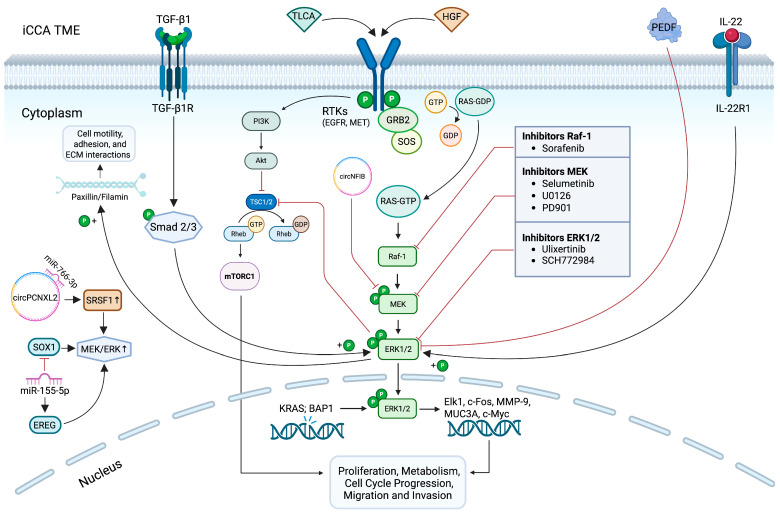
Aberrant activation of the ERK1/2 signaling cascade in iCCA and its therapeutic targeting. Extracellular and intracellular stimuli can induce the hyperactivation of the ERK1/2 signaling cascade. Upon stimulation, RAS recruits and activates Raf-1 kinase, which phosphorylates MEK1/2, subsequently leading to the activation of ERK1/2 in the cytoplasm. Once phosphorylated, ERK1/2 translocates to the nucleus and regulates multiple downstream effectors involved in a wide range of cellular processes. Dysregulation of this pathway contributes to tumor growth and progression in iCCA, highlighting ERK1/2 as a promising target for molecular therapy. The figure illustrates the main components of this pathway and the small-molecule inhibitors currently under investigation to counteract aberrant ERK1/2 activation in iCCA. It also depicts the compensatory activation of the PI3K/AKT/mTORC1 pathway in response to ERK1/2 inhibition. It was created with BioRender (https://biorender.com/, accessed on 19 June 2025). Symbols: P, phosphate group; GDP, guanosine diphosphate; GTP, guanosine triphosphate.

ERK1/2 is a member of the mitogen-activated protein kinase (MAPK) family, which plays a key role in activating a broad array of downstream targets involved in several cellular processes, including proliferation, survival, differentiation, apoptosis, and cell motility (Figure 1). All of these processes are fundamental to maintaining cellular plasticity under physiological conditions [31,33]. Activation of this signaling pathway initiates at the cell membrane, where a variety of extracellular ligands engage membrane receptors such as G-protein-coupled receptors (GCPRs), receptor tyrosine kinases (RTKs), and integrins, triggering a cascade of intracellular signaling events [32,34,35].

While ERK1/2 may be activated through alternative mechanisms such as Gq-PI3K-Raf-1 and AC-cAMP-PKA-Raf-1 [36,37] pathways, its canonical activation route is the Ras-Raf-1-MEK-ERK1/2 cascade, which involves a series of serine/threonine phosphorylation events [38].

Upon activation, Ras stimulates Raf-1, a serine/threonine kinase belonging to the MAP3K family. Raf-1, in turn, phosphorylates and activates the dual-specificity kinases of the MAP2K family (MEK1/2). MEK1/2 then propagate the signal by phosphorylating ERK1/2 on key tyrosine (Y204/Y187) and threonine (T202/T185) residues, leading to the full activation of ERK1/2 [39]. Dephosphorylated ERK1/2 is localized in the cytoplasm [40]. Upon activation, ERK1/2 rapidly translocates across the nuclear membrane, representing a critical step in multiple cellular regulatory functions [40]. Within the nucleus, ERK1/2 phosphorylates key transcription factors, such as c-Fos, Elk-1, and c-Myc, thereby facilitating the conversion of extracellular signals into specific gene expression programs [38,41]. These programs govern fundamental cellular processes, such as proliferation and survival, growth and metabolism, and cell cycle progression [33]. Nevertheless, upon activation, ERK1/2 also translocates to various other subcellular compartments, where it regulates a wide range of downstream targets. Specifically, active ERK1/2 translocation to mitochondria regulates cell fate, acting through the intrinsic survival-apoptotic pathway, predominantly controlled by the BCL2 protein family. ERK1/2 promotes cell survival by regulating pro-survival proteins such as BCL2, BCL-x, and MCL1, while concomitantly inhibiting pro-apoptotic proteins including BAD, BIM, and PUMA [42]. However, under nutrient deficiency, ERK1/2 can also induce the expression of the pro-apoptotic protein NOXA, suggesting its context-dependent role in shifting the balance between autophagy and apoptosis [43]. Additionally, ERK1/2 is involved in mitochondrial fission through phosphorylation of the GTPase dynamin-related protein 1 (DRP1) that forms higher-order ring structures that promote mitotic fission via GTP-dependent scission [44,45]. Another critical function of ERK1/2 concerns the regulation of Golgi apparatus fragmentation, a process recognized as an essential mitotic checkpoint. The inhibition of fragmentation by MEK1/2 inhibitors suggests that the ERK1/2 signaling cascade plays an essential role in its control [46]. Moreover, Golgi fragmentation has been correlated with the accumulation of monophosphorylated ERK1/2 within the organelle, further implicating ERK1/2 in the structural remodeling of the Golgi during cell division [47]. Furthermore, ERK1/2 is involved in mechanotransduction, whereby mechanical stimuli are converted by cells into biochemical signals that lead to structural and functional changes. This process involves myosin-driven tension on actin filaments anchored to cell–cell or cell–matrix adhesions and the activation of mechanosensitive proteins [48]. It has been observed that ERK1/2 directly or indirectly phosphorylates structural proteins such as paxillin [49] and filamin [50]. This activity modulates cell motility, adhesion, and interactions with the ECM, which are vital for cell migration and response to mechanical stimuli. In integrin outside-in signaling, the RIAM-VASP complex mediates the integrin complement receptor, facilitating particle engulfment and modulating ERK1/2 phosphorylation kinetics. Concomitantly, β1 integrin/FAK/cortactin signaling, together with ERK1/2 and JNK1/2 pathways, regulates focal adhesions disassembly and turnover, thus promoting cell survival [51,52].

Given its involvement in numerous essential cellular processes, the dysregulation of ERK1/2 signaling can have detrimental cellular effects. Persistent hyperactivation of the ERK1/2 cascade is a hallmark of various cancers, where its constitutive activation drives tumorigenesis by promoting uncontrolled proliferation, enhanced survival, increased migratory and invasive capacity, and extensive remodeling of the ECM. These effects contribute to the aggressive behavior of the tumor and its resistance to most conventional therapies [53].

While ERK1 and ERK2 isoforms are often considered functionally interchangeable in this oncogenic context, increasing evidence suggests that they may exert distinct biological roles. Notably, the ERK1 and ERK2 share approximately 85% of amino acid sequence identity [54]. Despite their high homology, several studies debate how the two isoforms, although activated by similar upstream stimuli, are not entirely redundant under physiological conditions. Differences between them have been observed in terms of cellular localization, expression patterns, and biological function [54]. In mast cells, it was observed that ERK1 and ERK2 exhibit distinct subcellular distributions following stimulation, suggesting isoform-specific roles [55]. ERK1, once activated, tends to translocate rapidly into the nucleus, modulating gene expression by mediating the activity of transcription factors. Conversely, ERK2 predominantly localizes in the pericentrosomal region, indicating its potential involvement in cytoskeleton organization [55]. Further evidence of functional divergence comes from studies in pancreatic beta cells, where ERK1 is required for the full activation of mitogen- and stress-activated protein kinase 1 (MSK1) and cAMP response element-binding protein (CREB), two key molecules involved in glucose metabolism [56]. Notably, the presence of ERK2 alone cannot compensate for the absence of ERK1 in this context, underscoring their non-interchangeable and distinct functional roles [56]. On the contrary, in fibroblasts, the hypothesis of functional redundancy between ERK1 and ERK2 is supported, as both isoforms have been shown to sustain cell proliferation equally [57,58].

The potential functional differences between ERK1 and ERK2 have long been debated in cancer research. Many studies have found that ERK2 has a predominant role in tumorigenesis and cancer progression [59,60,61].

In hepatocyte growth factor (HGF)-stimulated non-small-cell lung cancer (NSCLC) cells, ERK2 silencing, but not ERK1, significantly impairs tumor cell motility [59]. In ERK-driven tumors, such as *BRAF*-mutant melanoma, cancer cells show a marked dependence on ERK2, with its inhibition leading to a substantial decrease in proliferation markers, an effect that is not reproduced by ERK1 silencing [60]. Similarly, in HCC, ERK2 RNA interference (RNAi) experiments on the HuH7 lineage demonstrated complete inhibition of cell proliferation in vitro and tumor growth in vivo, while ERK1 silencing did not have the same effect. Moreover, ERK2 has been shown to promote cell motility and invasiveness via the urokinase plasminogen activator (uPA)/p70S6K pathway, a mechanism not replicated by ERK1 [61]. These findings indicate that, under certain oncogenic contexts, ERK2 may exert functions not compensated by ERK1.

## 3. Role of ERK1/2 Signaling in iCCA Tumorigenesis and Progression

**Figure 2:**
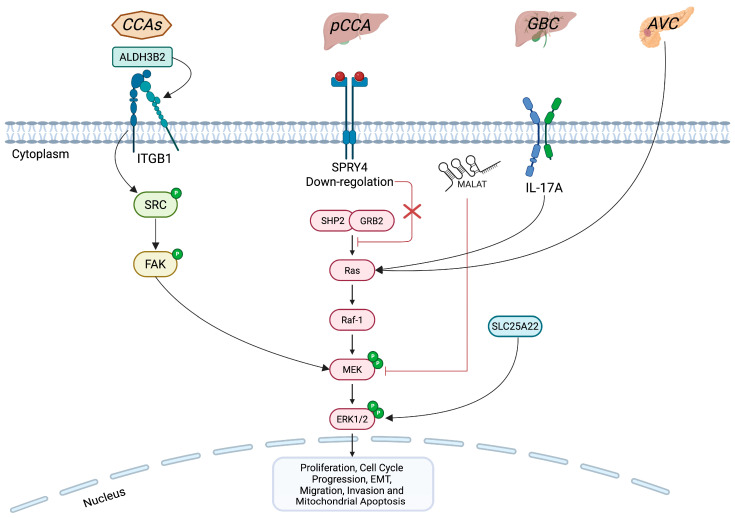
Comparison of ERK1/2 signaling impairment in CCAs versus other biliary tract cancers. The figure highlights key effectors involved in the altered regulation of the ERK1/2 pathway across biliary tract tumors. In all three CCA (CCAs), ALDH3B2 via ITGB1 drives ERK1/2 hyperphosphorylation. In GBC, IL-17A, the mitochondrial glutamate carrier SLC25A22 and the non-coding RNA MALAT1 contribute to aberrant ERK1/2 activation, fostering malignant progression. AVC also exhibits ERK1/2 pathway overactivation. Conversely, SPRY4 is downregulated in pCCA, but, when highly expressed, it acts as an endogenous inhibitor of ERK1/2 phosphorylation, thereby limiting tumor proliferation. The figure was created with BioRender (https://biorender.com, accessed on 19 June 2025). Symbols: P, phosphate group.

iCCA is one of three main subtypes of cholangiocarcinoma (CCA), alongside perihilar (pCCA) and distal (dCCA) forms. However, ERK1/2 pathway regulation has been reported across all three CCA, as well as in other biliary tract cancers, such as gallbladder cancer (GBC) and in the adenocarcinomas of the ampulla of Vater (AVC) (Figure 2) [7,62,63,64,65,66,67].

A recent study highlighted the pro-oncogenic role of the aldehyde dehydrogenase 3 family member B2 (ALDH3B2) enzyme in the three subgroups of CCA through the molecular axis ALDH3B2–integrin β1 (ITGB1)–ERK1/2 [68]. Specifically, ALDH3B2 via ITGB1 leads to an increase in the phosphorylation rate of ERK1/2, promoting tumor growth and the invasion and metastasis of CCA cells (Figure 2) [68]. However, given the marked molecular heterogeneity among CCA, it is conceivable that ERK1/2 activation arises from different upstream drivers in each group [69]. For instance, in iCCA, *FGFR2* fusions lead to the ligand-independent activation of ERK1/2, promoting cell proliferation [70]. Additionally, *KRAS* mutations in iCCA also activate ERK1/2 signaling, which contributes to immune evasion by stabilizing programmed death-ligand 1 (PD-L1) and preventing its autophagy-mediated degradation [20]. A recent omics study employing phosphoproteomic analyses of primary iCCA tumors and established iCCA cell lines has consistently demonstrated elevated levels of ERK1/2 phosphorylation, underscoring its pivotal role as one of the drivers of iCCA development [71]. Conversely, in pCCA subtype, it has been shown that Sprouty RTK signaling antagonist 4 (SPRY4) is significantly downregulated in tumor tissue compared to adjacent non-tumor tissue, and that its low expression correlates with worse prognosis [72]. Molecularly, SPRY4 acts as an endogenous inhibitor of the FGFR2-ERK1/2 axis. When SPRY4 is expressed at high levels, it blocks ERK1/2 phosphorylation, leading to reduced tumor proliferation and inducing G1-phase arrest (Figure 2) [72].

Although these findings underscore the central role of the ERK1/2 pathway in CCA tumorigenesis, most of the scientific evidence is focused on iCCA, where the aberrant activation of ERK1/2 signaling has been reported by several external and internal stimuli.

### 3.1. RTKs: HGF/Met and TLCA/EGFR Axes

Over the past decade, many studies have demonstrated that various upstream regulators within the TME contribute to the hyperactivation of ERK1/2 observed in iCCA. Among these, hepatocyte growth factor (HGF) plays a prominent role by promoting tumor invasion through the disruption of the cell–cell adhesion complex, cell adhesion to the ECM, cell motility, and the production of matrix-degrading enzymes, such as MMPs and uPA [73]. HGF exerts its effects through binding to the tyrosine kinase receptor Met, involved in tumor progression, including iCCA (Figure 1) [74]. Stimulation of Met triggers the activation of multiple downstream signaling cascades, predominantly the MAPK/ERK1/2 and PI3K/Akt signaling axes, which are critically involved in promoting tumor cell proliferation, survival, and invasiveness [75]. Gene expression studies demonstrated that Met is overexpressed in approximately 20–60% of patients with iCCA. This overexpression is frequently correlated with poor clinical outcomes, highlighting its potential as both a prognostic biomarker and a therapeutic target [76]. In one study, HGF induced invasion and motility of two human iCCA cell lines, HuCCA-1 and KKU-M213, both of which overexpressed Met [74]. Interestingly, while PI3K signaling was essential for HGF-induced invasiveness in both lines, the importance of ERK1/2 in promoting tumor invasion was related to the duration of ERK1/2 activation. In KKU-M213 cells, HGF-induced invasion is driven by a prolonged activation of ERK1/2, whereas in HuCCA-1 cells, HGF only triggers a rapid and transient ERK1/2 activation, which appears to be dispensable to promote invasion. These findings suggest that the temporal dynamics of ERK1/2 signaling may play a critical role in modulating the invasive potential of iCCA cells [74]. Furthermore, Vanichapol and colleagues reported that hypoxic stress selectively increased ERK1/2 phosphorylation without affecting Akt, enhancing the aggressive phenotype of iCCA cells by increasing their invasive capabilities through Met activation and ERK1/2 hyperphosphorylation [77].

Among the various components of bile acids, taurolithocholic acid (TLCA) is considered to be another effector promoting the increase in ERK1/2-phosphorylated in iCCA (Figure 1).

It has been demonstrated that TLCA induces the activation of the ERK1/2 signaling pathway via epidermal growth factor receptor (EGFR), thus promoting iCCA proliferation [78].

### 3.2. TGF-β1

Strikingly, it has been hypothesized that ERK1/2 is involved in the resolution of the transforming growth factor beta (TGF-β1) paradox, a phenomenon in which TGF-β1 functions as a tumor suppressor during the early stages of carcinogenesis by inhibiting cell proliferation and inducing apoptosis, but later shifts to promoting cancer cell invasion and spreading [79]. Since both TGF-β1 and ERK1/2 are involved in iCCA invasion [74,80], Sritananuwat and colleagues proposed that ERK1/2 signaling may play a key role in mediating this functional switch [21]. Specifically, it has been shown that, in iCCA cell lines, TGF-β1 enhances cell migration and invasion by inducing ERK phosphorylation via the Smad pathway at the expense of cell proliferation (Figure 1). Blocking ERK1/2 overactivation using the MEK1/2 inhibitor U0126 impairs the pro-invasive effects of TGF-β1 while restoring its antiproliferative tumor-suppressive function. These findings pave the way for targeting ERK1/2 as a promising strategy to selectively inhibit TGF-β-induced invasion and metastasis in iCCA cells, without impairing its cytostatic activity [21].

### 3.3. PD-L1/PD-1 Axis

Additionally, ERK1/2 is also crucial in the mechanisms by which the tumor evades immune surveillance [20]. One of the most effective strategies employed by cancer cells to escape immune detection is the interaction between the PD-1 receptor (expressed on T lymphocytes) and its ligand PD-L1 (expressed on tumor cells) [53,81]. It has been observed that, in iCCA cells harboring *KRAS*-activating mutations, phosphorylation of the Raf-1/MEK/ERK cascade promotes PD-L1 expression, thereby contributing to so-called “immune evasion” [20]. Zheng Gao et al. highlighted that ERK1/2 inhibition, via the potent and selective ERK1/2 inhibitor SCH772984, not only reduces PD-L1 levels, but also induces autophagy. This process leads to the degradation of PD-L1 and makes tumor cells more vulnerable to attack by activated CD8+ T lymphocytes. Specifically, researchers observed an increase in autophagy markers, such as LC3-II, Beclin-1, and ATG5, and a reduction in the level of p62/SQSTM1, confirming the activation of the process. This translated into more effective immune activity against the tumor [20]

### 3.4. PEDF

In a previous study, we demonstrated that within the iCCA TME, the matricellular proteins thrombospondin-1 (THBS1) and thrombospondin-2 (THBS2), and the pigment epithelium-derived factor (PEDF), a multifunctional soluble glycoprotein, are highly expressed and secreted by both cancer-associated fibroblasts (CAFs) and tumor cells [82].

These proteins act as direct and indirect key regulators of the iCCA progression by promoting the tumor-associated lymphangiogenesis and influencing the iCCA growth and metastatic dissemination. However, whereas the exogenous administration of recombinant THBS1 and THBS2 has been shown to promote iCCA cell proliferation, migration, invasion, and adhesion [83], the paracrine activity of PEDF impairs iCCA cell motility and reduces ERK1/2 phosphorylation, thus suggesting PEDF as a new upstream regulator of the MAPK/ERK signaling pathway in iCCA (Figure 1) [84]. Further efforts aimed at identifying the iCCA cell surface receptor engaged by PEDF may gain insights into the most specific and valuable target able to inhibit the aberrant activation of ERK1/2 signaling pathway and counteract iCCA cell spreading. Although PEDF-based therapies targeting the ERK1/2 pathway are not currently available, a recent study from a breast cancer model suggests a role of PEDF in downregulating tumor-promoting factors influenced by ERK1/2 activation. It has been observed that PEDF, when combined with the chemotherapeutic agent doxorubicin, exerts synergistic effects in reducing breast cancer cell migration and invasion [85]. Specifically, the synergy between PEDF and doxorubicin suppresses both the intracellular pro-metastatic signals induced by PI3K/AKT and p38-MAPK pathways and the expression of extracellular invasion-related factors, including uPA, CXCR4, and MT1-MMP [85]. Although ERK1/2 was not the primary target in that study, the modulation of multiple MAPK components suggests a potential crosstalk.

### 3.5. IL-22/IL-22R1 Axis

The IL-22/IL-22R1 signaling axis has been commonly associated with the development and progression of numerous cancers, including gastrointestinal malignancies, by activating several signaling pathways, including the MAPK/ERK1/2 [86]. A recent study has shown that the IL-22R1 receptor is overexpressed in human iCCA tissues and cell lines (i.e., HuCCT-1 and RBE) [75]. Upon binding to its receptor, IL-22 enhances iCCA cell mitosis, promoting proliferation, migration, invasion, and resistance to apoptosis through ERK1/2 phosphorylation. Notably, the selective inhibition of ERK1/2 by SCH772984 markedly suppresses IL-22/IL-22R1-induced ERK1/2 phosphorylation in both in vivo and in vitro models (Figure 1) [75].

### 3.6. BAP1

It is known that breast cancer type 1 susceptibility protein-associated protein-1 (BAP1) is a broad-spectrum tumor suppressor in many cancer types [87]. Chen et al. have identified BAP1 as a potential tumor suppressor in iCCA by negatively modulating the ERK1/2 pathway (Figure 1) [88]. They demonstrated that BAP1 is frequently downregulated in human iCCA tissues and cell lines (i.e., HCCC9810 and RBE). Loss of BAP1 led to an increase in ERK1/2 phosphorylation and signaling activity, thereby promoting iCCA progression. The tumor-suppressive function of BAP1 has been further supported by antagonizing the activity of the ERK1/2 signaling pathway with the U0126 inhibitor, which reduced iCCA cell proliferation, cell cycle progression, and invasion [88].

### 3.7. Non-Coding RNA Regulation

The regulation of the ERK1/2 pathway in iCCA is further complicated by the involvement of non-coding RNAs, such as microRNAs (miRNAs) and circular RNAs (circRNAs). These molecules can exert opposing effects, some acting as tumor suppressors, while others contribute to tumor progression.

circRNAs represent a kind of novel RNA transcripts, which are generated by back-splicing events that covalently link a downstream 5′ splice site to an upstream 3′ splice site. Increasing evidence shows that circRNAs are important regulatory molecules that play crucial roles in the development and progression of many diseases, including iCCA [89,90]. The most frequently reported mechanism of action is their ability to act as microRNA (miRNA) sponges, thereby modulating miRNA availability and indirectly regulating target gene expression. Nevertheless, additional functions are emerging, such as transcriptional and post-transcriptional regulation, protein scaffolding, and even peptide translation in some contexts. miRNAs are short non-coding RNAs that modulate gene expression, primarily at the post-transcriptional level, by binding the 3′ UTR of mRNAs, thereby inhibiting translation or promoting mRNA degradation [91]. Due to their covalently closed structure, circRNAs are resistant to exonuclease-mediated degradation, which contributes to their remarkable stability compared to linear RNAs. Such intrinsic physical features mean that they could be promising diagnostic and prognostic biomarkers [92]. In addition, circRNAs are found circulating in liquid biopsies, such as blood, urine, saliva, and bile, either freely or encapsulated within Extracellular Vesicles (EVs) [93]. Moreover, detection methods for circRNAs, particularly RT-qPCR with primers designed to span the unique back-splice junction, tend to offer greater specificity and sensitivity compared to conventional immunoassays used for protein-based markers [91]. There is growing evidence showing that circRNAs are deregulated in cancer and contribute to the regulation of oncogenic processes. Thus, circRNAs, as well as miRNAs, might be considered as relevant therapeutic targets in cancer [94]. Several strategies have been proposed to inhibit oncogenic circRNAs, such as antisense oligonucleotides (ASOs), LNA gapmers targeting the back-splice junction, and CRISPR/Cas13 systems. Other approaches aim to modulate circRNA biogenesis by interfering with splicing factors or intronic complementary sequences. Conversely, synthetic circRNAs are being developed to sequester onco-miRNAs or proteins, or even to express tumor suppressor proteins. Given their stability and versatility, circRNA-based therapies hold strong potential in oncology and are supported by existing RNA-based drug platforms [94].

#### 3.7.1. Tumor-Suppressive Non-Coding RNA

In an iCCA scenario, a recent study demonstrated that the circRNA *CircNFIB* (hsa_circ_0086376) is frequently downregulated in human iCCA tissues and that its loss promotes tumor invasion and metastasis, as shown in both in vitro and in vivo models (Figure 1) [95]. Their findings suggest that *CircNFIB* may function as a natural suppressor of tumor progression in iCCA by directly binding to MEK1 and preventing its interaction with ERK1/2, thereby inhibiting activation of the ERK1/2 signaling pathway [95].

#### 3.7.2. Oncogenic Non-Coding RNA

On the contrary, *circPCNXL2* (hsa_circ_0016956) is overexpressed in iCCA and promotes tumor cell proliferation and metastasis by interacting with serine-threonine kinase receptor-associated protein (STRAP) and, consequently, facilitating the activation of MEK1/2-ERK1/2. Additionally, *circPCNXL2* acts as a competing endogenous RNA by sponging miR-766-3p, leading to the upregulation of SRSF1, another known activator of ERK1/2 signaling (Figure 1) [96].

Tang et al. demonstrated that miR-155-5p, significantly upregulated in iCCA, directly targets epiregulin (EREG) [97], a member of the epidermal growth factor (EGF) family involved in cell proliferation, differentiation [98], and stemness [99]. Through this interaction, miR-155-5p promotes EGFR activation and the subsequent stimulation of the MEK/ERK1/2 cascade. This results in the increased phosphorylation of MEK1/2 and ERK1/2, thus promoting cell survival [97]. Additionally, miR-155-5p is implicated in the regulation of *SOX1* expression. Notably, *SOX1* is downregulated in iCCA, and its reduced expression is associated with enhanced activation of the ERK1/2 signaling pathway [100]. It has been demonstrated that miR-155-5p may function as an oncogenic miRNA directly targeting the 3′UTR of *SOX1* mRNA, leading to its post-transcriptional repression. The resulting downregulation of *SOX1* contributes to the reactivation of the Raf-1/MEK/ERK1/2 signaling cascade, thereby contributing to the oncogenic progression of iCCA [100].

### 3.8. ERK1/2 Downstream Targets

In iCCA, the abovementioned hyperactivation of the ERK1/2 signaling pathway directly engages several downstream effectors involved in promoting cell proliferation, invasiveness, survival, and immune evasion. Sritananuwat et al. demonstrated that TGF-β1-induced ERK1/2 activation promotes the secretion of MMP-9, a matrix metalloproteinase responsible for extracellular matrix degradation, thereby enhancing the invasive and migratory capacity of KKU-M213 cells. The inhibition of ERK1/2 with U0126 led to reduced MMP-9 expression and decreased cellular invasiveness, supporting the direct role of ERK1/2 in regulating iCCA tumor invasion [21].

A more recent study revealed that ERK1/2 also contributes to immune privilege acquisition in CCA cells. Specifically, the interaction between 67LR and laminin activates ERK1/2, which in turn phosphorylates and activates c-Myc at serine 62. Activated c-Myc upregulates FasL transcription in tumor cells, enabling them to induce apoptosis in Fas-expressing immune cells. Notably, inhibition of ERK1/2 using PD98059 or c-Myc silencing by siRNA significantly reduced FasL expression and impaired the tumor ability to induce apoptosis in Fas-sensitive Jurkat T cells [101].

Furthermore, Baijie Feng et al. highlighted the involvement of the MAPK/ERK1/2 pathway in the upregulation of mucin 3A (MUC3A) in iCCA. ERK activation stimulates MUC3A expression, which promotes cancer cell proliferation, invasion, and migration by modulating the cell cycle and epithelial–mesenchymal transition [102].

Collectively, these findings highlight the pivotal role of ERK1/2 signaling in iCCA tumorigenesis and progression, underscoring its potential as a therapeutic target.

## 4. Unraveling the Potential Role of ERK1/2 Signaling in iCCA Targeted Therapy

As previously mentioned, iCCA is marked by pronounced inter- and intra-tumoral heterogeneity, along with a distinct TME that is highly desmoplastic and poorly vascularized. These features pose significant challenges not only in the identification of reliable biomarkers for early diagnosis, but also in developing effective treatments, thereby limiting the available therapeutic options [103,104]. Most iCCA patients with advanced, unresectable, and metastatic disease receive a palliative systemic therapy, with a combination of gemcitabine and cisplatin as first-line treatment and FOLFOX as second-line standard of care [105]. However, with the advent of PM and TT, the therapeutic approach for iCCA patients is gradually shifting toward molecularly targeted strategies.

In this new therapeutic scenario, targeted inhibition of the ERK1/2 pathway, which is hyperactive in at least 40% of cancers and plays a pivotal role in tumor cell proliferation and metastasis, is an established strategy in various tumors [32]. Accordingly, several MEK1/2 inhibitors have been tested in preclinical and clinical models [17,106]. As previously discussed, ERK1/2 hyperactivation plays a pivotal role in the tumorigenesis and progression of iCCA. Although current evidence does not support ERK1/2 as a biomarker for early diagnosis, its central involvement in iCCA pathogenesis highlights it as a promising therapeutic target for developing TT. Currently, the main goal is to inhibit the abnormal activation of the ERK1/2 cascade using small-molecule inhibitors, most of which are commonly used in other types of cancer [32]. Some of these compounds, summarized in Table 1 and shown in Figure 1, are now being evaluated in experimental models and clinical trials for iCCA.

In a recent study by Schüler et al., the authors investigated the effects of the MEK1/2 inhibitor, Selumetinib, on three established iCCA cell lines: HuH28, RBE, and SSP25. It has been demonstrated that Selumetinib significantly reduces the rate of ERK1/2 phosphorylation, leading to an inhibition of the iCCA cell proliferation [107].

**Table 1:** ERK1/2 small-molecule inhibitors evaluated in preclinical and clinical studies for iCCA management.

Small- Molecule Inhibitors	Target	Features in iCCA Cells	Status in iCCA	AUC (0–t) (ng·h/mL)	AUC (0–∞) (ng·h/mL)	C_max_ (ng/mL)	T_max_ (h)	Resistance	Ref.
**Selumetinib**	MEK1/2	Inhibition of cell proliferation	Preclinical and clinical	4757.16	5653.31	1331.33	1.55		[108,109,110]
**U0126**	MEK1/2	Inhibition of cell proliferation, cell cycle progression, and invasion	Tested in vitro and in vivo						[88,109]
**PD901**	MEK1/2	Increased apoptosis, inhibition of cell proliferation	Tested in vitro and in vivo						[109]
**Sorafenib**	Raf-1	Cell cycle arrest, increased apoptosis	Preclinical and clinical	18,000–23,000	20,000–26,000	3000–4000	3–4	Activation of the PI3K/Akt pathway; Induction of CYP3A4 with prolonged sorafenib use	[111,112]
**Ulixertinib** **(BVD-523)**	ERK1/2	Cell cycle arrest, increased apoptosis	Preclinical and clinical	19,930,000	Not available due to measurement limitations (12 h sampling)	2120	2–4		[25]
**Selumetinib** **+** **MK-2206**	MEK and Akt	Inhibition of cell proliferation	Tested in vitro						[113]

Pharmacokinetic parameters include the following: area under the plasma concentration–time curve from time zero to the last measurable concentration (AUC₀–t, ng·h/mL); area under the plasma concentration–time curve from time zero to infinity (AUC₀–∞, ng·h/mL); maximum observed plasma concentration (Cmax, ng/mL); and time to reach Cmax (Tmax, h).

Approximately 15–25% of iCCA patients harbor gain-of-function mutations in the *KRAS* gene, generally associated with poor prognosis [20]. Constitutive activation of *KRAS* leads to the hyperphosphorylation of the MEK/ERK1/2. This continuous signal propagation sustains pro-tumorigenic networks that drive the growth and survival of cancer cells [108].

In a 2018 study, the MEK1/2 inhibitors U0126, PD901, and Selumetinib were shown to significantly reduce ERK1/2 phosphorylation, thereby suppressing the proliferation of iCCA cells in vitro [109]. In the same study, the authors developed a novel *KRAS*-driven murine model of iCCA to further explore the mechanisms of tumorigenesis and evaluate the therapeutic efficacy of targeting the ERK1/2 signaling pathway. Given that U0126 requires relatively high concentrations to exert its inhibitory effects in vitro and is not suitable for in vivo applications due to limited pharmacokinetic properties [114], the study focused on PD901 for in vivo evaluation. Indeed, PD901 was specifically engineered to achieve high membrane permeability and favorable systemic bioavailability [115], making it a more suitable candidate for preclinical studies. Administration of PD901 in the *KRAS*-driven iCCA mouse model resulted in a marked increase in apoptosis within tumor tissues. At the molecular level, PD901 effectively inhibited ERK1/2 activation, confirming the critical role of the MEK/ERK signaling axis in sustaining iCCA cell survival and highlighting the potential of MEK inhibition as a therapeutic strategy [109].

Another potential therapeutic approach involves the use of Sorafenib, a multi-kinase inhibitor approved for the treatment of various solid tumors, including HCC and renal cell carcinoma [34,116]. Sorafenib exerts its antitumor activity by inhibiting Raf-1, thereby blocking the MAPK cascade and preventing ERK1/2 phosphorylation. This results in cell cycle arrest and apoptosis in cancer cells [117]. However, despite its mechanism of action, Sorafenib has not yet proven to be effective for iCCA treatment. For instance, Jan-Paul Gundlach and colleagues demonstrated that Sorafenib’s efficacy is highly dependent on the TME context. They observed that, in co-culture assays with iCCA cells and CAF or Schwann cells (SCs), treatment with Sorafenib caused a reduction in CAF migration capacity, but not in SCs, providing a possible explanation for why Sorafenib failed to treat patients with iCCA in previous clinical trials [111]. Overall, these findings underscore that the inhibition of ERK1/2 signaling pathway still faces major challenges due to suboptimal Sorafenib properties and the heterogeneous nature of the TME. Addressing these barriers will require the development of more selective and efficacious compounds, deeper insights into tumor–TME interactions, and the implementation of combinatorial therapeutic strategies to overcome resistance and improve patient outcomes. In the same year, another study aimed at standardizing the label-free imaging analysis to monitor the tumor growth of patient-derived iCCA organoids (iCCAO) following drug treatment. The data revealed that iCCAO growth was inhibited by Sorafenib in a time/dose-dependent manner, while non-tumor intrahepatic cholangiocyte organoids were not affected [112].

### 4.1. ERK1/2 Pathway Inhibition Strategies in ERK1/2-Driven Cancers vs. Icca: Clinical Perspectives and Emerging Evidence

Although the mechanisms underlying overactivation of the ERK1/2 pathway across different tumors share strong similarities, such as mutations in *KRAS*, *BRAF*, or FGFR2 fusions, it is crucial to consider the extent to which a tumor truly depends on ERK1/2 signaling to sustain its continuous growth and survival [118]. In this context, Bernard Weinstein coined the term “oncogene addiction”, which describes how cancer cells, despite harboring numerous genetic and epigenetic abnormalities, can become dependent only on a single pro-tumor pathway activated to ensure the maintenance of their malignant phenotype [119]. This concept becomes crucial for TT, as the development of inhibitors directed against specific targets can be pivotal for designing monotherapy or dual-targeted therapeutic approaches. Phase I clinical studies have shown that ERK1/2 small-molecule inhibitors may have therapeutic potential as monotherapy, depending on the degree of oncogene addiction to the ERK1/2 pathway [25,120]. An example is given by *BRAF*-mutant melanoma, where ERK1/2 is considered to be the key oncogenic driver [60,121]. Regarding this, a Phase I clinical study assessed the therapeutic efficacy of the drug Ulixertinib (BVD-523) based on its safety, pharmacokinetics, and antitumor activity in patients with advanced solid tumor [25]. Specifically, Ulixertinib is an ATP-competitive inhibitors of ERK1/2 that selectively and reversibly inhibits ERK1/2, leading to cell cycle arrest and apoptosis in tumor cells [122]. Sullivan and colleagues [25], in a clinical Phase I study performed on advanced solid tumor, including iCCA, demonstrated that Ulixertinib can be considered a potential monotherapy drug in tumors with *BRAF*-mutant melanoma. Notably, Ulixertinib reduced or blocked tumor growth in patients with resistance to prior BRAF/MEK-targeted inhibitors (e.g., Vemurafenib or Trametinib), as well as in those harboring non- *BRAF* mutations. Furthermore, this ERK1/2 small-molecule inhibitor showed promising efficacy with a favorable toxicity profile, leading to the identification of a recommended Phase II dose, which achieved the near-complete inhibition of ERK signaling at therapeutically relevant concentrations. Conversely, no significant monotherapy efficacy has been reported in the iCCA patient cohort, possibly due to limited therapeutic activity [25]. Another Phase 1b clinical trial reported that the selective ERK1/2 inhibitor, MK-8353, demonstrated substantial therapeutic efficacy both as monotherapy and in combination treatments in BRAF V600-mutated melanoma. In contrast, efficacy was limited in tumors such as *KRAS*-mutated NSCLC, likely because these tumor cells are not addicted to ERK1/2 signaling or can compensate for its inhibition via alternative pathways (NCT02972034) [120]. These findings highlight the importance of understanding the primary oncogenic drivers sustaining the malignant phenotype, as such knowledge can guide the selection of monotherapy versus combination therapeutic strategies. In the case of iCCA, given its high intra-tumor heterogeneity and complex pathobiology, it cannot be defined as oncogene-addicted to ERK1/2 hyperactivation in the same way as *BRAF*-mutant melanoma. This supports a more rational shift toward combination approaches in iCCA treatment. Early clinical attempts using ERK1/2 small-molecule inhibitors for iCCA are summarized in Table 2.

Regarding Ulixertinib, it remains under investigation in the iCCA context. In a Phase I clinical study, Ulixertinib is being investigated within a compassionate program enrolling patients with solid tumors, including a small cohort of iCCA patients, harboring MAPK pathway alterations and exhibiting incomplete responses to standard therapies (NCT04566393) [26]. However, no specific outcome data have been reported as response or duration, likely due to a lack of significant clinical responses [26].

In another Phase Ib clinical trial (NCT03454035) [127], Ulixertinib was administered in combination with Palbociclib, a CDK4/6 inhibitor. In this study, among the various solid tumors analyzed, one iCCA patient achieved stable disease, suggesting potential clinical activity [127]. Additionally, a Phase II clinical study (NCT05221320) [27] is underway, evaluating Ulixertinib combined with hydroxychloroquine, an autophagy inhibitor, in patients with advanced gastrointestinal malignancies harboring MAPK pathway mutations, including iCCA. This “basket” trial design groups patients by cancer type, with all groups receiving the same drug combination. For the iCCA cohort, the study started with a small number of 13 patients. If at least four of them show a partial or complete tumor response, the cohort will be expanded for further analysis. Although the results are still pending, the inclusion of a larger iCCA patient population than in previous trials suggests that this combination strategy may hold therapeutic potential [27]. Overall, TT aimed at inhibiting ERK1/2 signaling pathway represents a promising and innovative strategy for developing more effective and personalized treatments for iCCA. However, substantial challenges related to drug selectivity and acquired resistance must be addressed to achieve meaningful improvements in iCCA patient outcomes.

### 4.2. Challenges in ERK1/2 Small-Molecular Inhibitors Therapy: Resistance Mechanisms, Toxicity Management, and Delivery Approaches

The use of small-molecular inhibitors as monotherapy often yields limited survival benefits, and is frequently associated with the development of therapeutic resistance [113].

In the context of TT directed at the MEK/ERK1/2 axes, the reactivation of ERK1/2 may occur in tumor cells, despite the use of selective inhibitors, allowing for a rapid recovery of alternative or MAPK pathway signaling and subsequent treatment escape supporting proliferation and survival of the tumor. In iCCA, the inhibition of the MEK/ERK pathway may induce the compensatory activation of the PI3K/AKT/mTORC1 pathway (Figure 1), as observed in other solid tumors [128,129].

It has been reported that MEK inhibition promotes the Akt phosphorylation reciprocal feedback loop between the two pathways [130]. In this regard, Ewald et al. demonstrated that the combined inhibition of the PI3K-AKT-mTORC1 and MEK/ERK1/2 pathways by small-molecular inhibitors, such as AKT inhibitor MK-2206 or MEK inhibitor AZD6244, on iCCA cells results in a strong synergistic effect on iCCA cell proliferation and survival. Notably, acquired resistance to AZD6244 was effectively reversed through combination treatment with MK-2206, underscoring the potential of multi-targeted strategies to overcome resistance mechanisms [113]. Concomitantly, another key mechanism of resistance is the reactivation of ERK signaling despite the presence of inhibitors. In particular, the amplification or mutation of upstream components of the cascade, such as KRAS or BRAF, can restore MAPK pathway activity. Little et al. investigated these mechanisms by generating AZD6244-resistant subpopulations from two human colorectal carcinoma cell lines: Colo205 (BRAF^V600E mutant) and HCT-116 (KRAS^G13D mutant) 131]. In both lines, resistant cells showed a marked increase in MEK and ERK phosphorylation compared to parental cells, suggesting that upstream oncogenic hyperactivation may contribute to the maintenance of ERK signaling, even under conditions of pharmacological inhibition [131]. These results highlight how tumor adaptation to the MAPK pathway blockade can occur through several mechanisms, including the following: the activation of parallel survival pathways (e.g., PI3K/AKT/mTORC1), the upregulation of RTKs such as EGFR or FGFR, and the direct reactivation of the MEK/ERK cascade through upstream genetic alterations. Understanding these mechanisms supports combined and adaptive therapeutic approaches aimed at preventing or overcoming resistance to targeted treatments.

Another important aspect to consider in the development of new therapies for the management of iCCA is the toxicological profile of ERK1/2 small-molecule inhibitors, which requires appropriate clinical monitoring. Aforementioned drugs, such as Selumetinib, Sorafenib, and Ulixertinib, although showing therapeutic potential, are also the focus of clinical studies that are investigating their possible side effects in order to develop optimal dosing strategies for patient outcomes. The reported adverse events associated with these agents are summarized in Table 2. A multicenter Phase II trial involving 28 patients with metastatic iCCA reported a partial response to Selumetinib in 12% and stable disease in 68% of cases for at least 16 weeks. Median progression-free survival (PFS) was 3.7 months, and median OS was 9.8 months. The most common adverse events included rash (90%), xerostomia (54%), and nausea (51%), mostly grade 1 or 2. Only 14% of patients required dose reductions due to toxicities such as fatigue, diarrhea, rash, or cellulitis (NCT00553332) [123]. However, another randomized Phase II study comparing Selumetinib combined with cisplatin and gemcitabine (CisGem) versus CisGem alone did not demonstrate significant improvements in PFS or OS. Furthermore, the addition of Selumetinib increased toxicity, with more patients in the selumetinib arms requiring chemotherapy dose reductions (NCT01242605) [110]. Regarding Sorafenib was assessed in a pilot trial with 44 patients with unresectable and advanced iCCA. Treatment resulted in a 12-week disease control rate of 15.9%, median PFS of 3.2 months, and median OS of 5.7 months. Grade 1–2 adverse events occurred in 75% of patients, with one case of severe grade 4 cutaneous toxicity. Sorafenib, combined with best supportive care, showed an acceptable safety profile in patients with unresectable, advanced iCCA [124]. In another single-center study, patients treated with sorafenib (400 mg twice daily) experienced primarily grade 1–2 toxicities, including skin rash, hand–foot syndrome, diarrhea, nausea/vomiting, elevated liver enzymes, and fatigue. One case of grade 3 hand–foot syndrome led to treatment discontinuation [125]. Lastly, the promising candidate Ulixertinib was associated with dermatologic adverse events (dAEs), which were among the most frequently reported, with an overall incidence of 79% (107/135) and combined skin rashes occurring in 76% (102/135) of patients. The most common dAEs were acneiform rash (33%), maculopapular rash (27%), pruritus (25%), and acneiform dermatitis (31%). Grade 3 events occurred in 19% (25/135) of patients, with no grade 4 or 5 events reported. The occurrence of at least one dAE was associated with stable disease, and acneiform rash correlated with partial tumor responses [25,126]. Given the limitations related to toxicity and suboptimal efficacy observed with current ERK1/2 inhibitors, innovative delivery systems, such as nanocarriers, offer a promising avenue to improve therapeutic outcomes. Nanocarriers are nanoscale colloidal systems (generally < 1000 nm) designed to deliver therapeutic agents to specific sites within the human body [132]. These systems can be composed of organic, inorganic, or hybrid materials, and their composition, size, shape, and surface characteristics can be tailored to optimize biodistribution, solubility, stability, and controlled drug release [133]. Their application in drug delivery provides several advantages. First, targeted delivery allows for the drug to be directed specifically to diseased cells or tissues, minimizing systemic side effects. Controlled release improves therapeutic efficacy, while enhanced solubility increases the bioavailability of poorly water-soluble drugs. Additionally, nanocarriers protect drugs from premature degradation, ensuring that the active agent remains intact until it reaches its target [133]. This innovative therapeutic strategy is currently being developed for the treatment of solid tumors. Specifically, nanoparticles with chemical and physical properties tailored to the TME are engineered to carry drugs directly to molecular targets [134]. This targeted delivery has the potential to reduce adverse effects, overcome resistance mechanisms, and ultimately improve the clinical management of iCCA. In this context, recent evidence reports the innovative advance in iCCA therapy. An example is given by Yang et al. (2021), who developed an innovative system based on functionalized graphene oxide nanosheets (GO-PEIs) as carriers for the delivery of four suppressive miRNAs (miR-194-5p, miR-125b-5p, miR-122-5p, and let-7c-5p) in the treatment of iCCA [135]. This nanocarrier improves miRNA transfection and stability, effectively reducing tumor growth and drug resistance both in vitro and in mouse models, with low toxicity. The results suggest promising therapeutic potential for the combination of gene and drug therapies in iCCA [135].

Although ERK1/2 pathway inhibitors, such as Selumetinib, U0126, PD901, Sorafenib, Ulixertinib, and AZD6244, are still in preclinical or early clinical stages for the treatment of iCCA, their delivery via nanocarriers has not yet been explored as a potential strategy. Nevertheless, nanocarrier-based approaches already investigated in iCCA may lay the groundwork for improving the targeted delivery and therapeutic efficacy of ERK-pathway inhibitors. In this regard, a recent study in *Advanced Science* by Xiwen Wu et al. [136] developed a non-viral gene therapy using nanoparticles for iCCA, aimed at restoring PBRM1 gene expression, whose loss is associated with cellular senescence and tumor progression. The spherical, uniform nanoparticles were suitable for intravenous administration and cellular endocytosis, allowing for safe and targeted gene delivery while avoiding the limitations of viral vectors [136]. Similarly, Dutta et al. [137] developed pH-sensitive nanoparticles for pancreatic ductal adenocarcinoma (PDAC), enabling the selective release of the ERK inhibitor SCH772984 in the hypoxic and acidic tumor microenvironment. These particles were made of PEG-b-poly(carbonate) block copolymers functionalized with tertiary amines to respond to acidic pH; they remain stable in circulation (pH 7.4), but destabilize in acidic tissues (e.g., tumors), ensuring targeted release and reducing systemic toxicity [137].

## 5. Conclusions

One of the major challenges in addressing the aggressiveness of iCCA is the development of effective strategies for early detection and, most critically, the implementation of targeted therapeutic interventions. Among the molecular mechanisms under investigation, the ERK1/2 signaling cascade has garnered increasing interest due to its pivotal role in cancer biology. Constitutive activation of this pathway is a hallmark in several types of cancer, driving tumorigenesis by promoting cell proliferation and survival, as well as facilitating the metastatic cascade marked by enhanced cancer cell adhesion, migration, and invasion. Emerging evidence has revealed that aberrant ERK1/2 activation is also a recurrent event in iCCA, contributing to its malignant phenotype [7,20].

In this review, we aimed to emphasize how diverse extra and intracellular stimuli can cause alterations in the ERK1/2 signaling pathway, thereby fostering tumor initiation and progression in the iCCA. Although the ERK1/2 cascade constitutes only a single node within the intricate network of signaling pathways underpinning iCCA pathobiology, recent advances in the development of chemical classes targeting ERK1/2 offer a promising therapeutic avenue.

In this context, to support and broaden the exploration of ERK1/2-targeted therapeutic strategies in the management of iCCA, we have outlined the most recent advancements from both experimental models and preclinical studies involving small-molecule inhibitors aimed at attenuating the hyperactivation of this signaling cascade. While these preliminary results are encouraging, several critical challenges must still be addressed. These include the emergence of drug resistance, an incomplete understanding of the molecular underpinnings of iCCA, and the marked inter- and intra-tumoral heterogeneity that complicates therapeutic efficacy and personalization. One of the disadvantages of small-molecule inhibitors targeting the ERK1/2 pathway lies in the emergence of resistance mechanisms. Evidence suggests that blocking the ERK1/2 cascade can trigger the compensatory activation of alternative oncogenic pathways such as PI3K-AKT [138], which are themselves frequently dysregulated in iCCA [28].

Given the intricate network of signaling pathways that sustain iCCA tumorigenesis and progression, future therapeutic strategies may rely on the use of ERK1/2 inhibitors that are not as isolated agents, but as partners within broader multi-targeted intervention frameworks. Such an approach would aim to simultaneously modulate multiple oncogenic signals, increasing the likelihood of durable clinical responses. Moreover, the advent of multi-omics approaches has revolutionized our understanding of iCCA by enabling a high-resolution stratification of patients into molecularly distinct subtypes, underscoring the profound inter- and intra-tumoral heterogeneity that characterizes this malignancy [18,139]. These cutting-edge methods have played a crucial role in advancing our understanding of iCCA pathogenesis and, in certain cases, have led to the discovery of novel therapeutic targets. For instance, the Food and Drug Administration (FDA) approved Ivosidenib in 2021 for patients with locally advanced or metastatic iCCA harboring an *IDH1* mutation [140]. Similarly, Pemigatinib and Futibatinib, pan-FGFR inhibitors, received accelerated approvals between 2020 and 2022 from both the FDA and European Medicines Agency (EMA) for advanced iCCA with *FGFR2* fusions or rearrangements [141]. Lirafugratinib, a selective and irreversible FGFR2 inhibitor, has demonstrated promising efficacy and reduced toxicity in early clinical trials [142,143]. Although initially effective, treatments with FGFR inhibitors in iCCA may induce “convergent tumor evolution”, whereby cancer cells bypass targeted inhibition by activating compensatory pathways that promote tumor growth and progression [144]. In a recent study, a retrospective analysis of 17 iCCA patients receiving FGFR inhibitors, such as Pemigatinib and Futibatinib, revealed that nearly 50% developed secondary mutations in MAPK-related genes, such as *KRAS* and *BRAF*, leading to the hyperactivation of the MAPK pathway, which compromised the inhibitory drug, encouraging tumor growth [145]. These findings underscore the need for a combination of therapeutic strategies aimed at mitigating tumor resistance mechanisms.

In this context, the ERK1/2 signaling axis has emerged as a compelling downstream target, particularly in cases where resistance to upstream inhibitors is driven by the reactivation of MAPK pathways. Concerning this, evidence suggests that ERK1/2 signaling, activated concomitantly with chemotherapeutic agents such as gemcitabine and cisplatin, may hinder the efficacy of anticancer treatment by suggesting a pro-tumorogenic or protective role against chemotherapy [146]. It has been observed that, when iCCA cells are exposed to gemcitabine and cisplatin, the combined pharmacological inhibition of ERK1/2 with U0126 increases the sensitivity of tumor cells to chemotherapy-induced death, indicating that the ERK1/2 pathway is involved in resistance mechanisms [146]. Even though therapeutic clinical advances are currently underway, we believe that, in this increasingly dynamic and complex scenario, TT directed against ERK1/2 in the context of iCCA should not be viewed as a therapeutic endpoint, but rather as components of the PM “toolkit”. In this evolving therapeutic landscape, the integration of artificial intelligence and multi-omics platforms offers powerful tools for tailoring interventions [147]. Given the high heterogeneity of iCCA, such technologies could enable the real-time adaptation of therapies, including ERK1/2-targeted approaches, with unprecedented specificity and clinical impact.

## Figures and Tables

**Table 2:** Clinical trials targeting ERK1/2 in iCCA.

Small- Molecule Inhibitors	Reference Number	Type of Study	Main Results	TRAEs	Ref.
Selumetinib	NCT00553332	Multicenter Phase II study	Partial response: 12% Stable disease: 68% PFS: 3.7 months OS: 9.8 months	Rash (90%), xerostomia (54%), nausea (51%), with dose reduction in 14% due to fatigue, diarrhea, and rash	[123]
**Selumetinib** + CisGem	NCT01242605	Randomized Phase II study	PFS: 6 months OS: 12 months	Increased toxicity requiring chemotherapy dose reduction in patients treated with Selumetinib	[110]
**Sorafenib**		Pilot study	DCR a 12 sett.: 15.9% PFS: 3.2 months OS: 5.7 months	Grade 1–2 toxicity: 75% 1 case of severe skin reaction (grade 4)	[124]
		Single-center study	OS: 5.7 months PFS: 5.5 months	Rash (33.3%), grade 3 hand-foot syndrome (6.7%)	[125]
**Ulixertinib** (BVD-523)	NCT01781429	Phase I	Stable disease for more than 6 months	dAEs in 79% Acneiform rash (33%), maculopapular rash (27%), pruritus (25%) Grade 3: 19% No Grade 4/5	[25,126]
**Ulixertinib** **(BVD-523)** **+** Palbociclib	NCT03454035	Phase Ib	1 iCCA patient achieved stable disease	Fatigue (70%), rash (62%), and nausea (54%) decreased lymphocyte count (77%), decreased WBC count (73%) and anemia (65%) (all grade) No Grade 4/5	[127]
**Ulixertinib** **(BVD-523)** **+** **Hydroxychloroquine**	NCT05221320	Phase II basket	Results still pending		[27]

The table reports treatment-related adverse events (TRAEs) and clinical trial endpoints, including the following: maximum tolerated dose (MTD), disease control rate (DCR), progression-free survival (PFS), and overall survival (OS).

## Data Availability

Not applicable.
